# Validity and reliability of the Baecke questionnaire against accelerometer-measured physical activity in community dwelling adults according to educational level

**DOI:** 10.1371/journal.pone.0270265

**Published:** 2022-08-15

**Authors:** William R. Tebar, Raphael M. Ritti-Dias, Rômulo A. Fernandes, Tatiana M. M. Damato, Mauro V. G. de Barros, Jorge Mota, Lars Bo Andersen, Diego G. D. Christofaro

**Affiliations:** 1 Post-graduation Program in Movement Sciences, Faculty of Sciences and Technology, São Paulo State University (Unesp), Presidente Prudente, Brazil; 2 Universidade Nove de Julho–Post-graduation program in Rehabilitation Sciences, Sao Paulo, Brazil; 3 Department of Physical Education, Universidade Federal de Pernambuco (UFPE), Recife, Brazil; 4 Research Center on Physical Activity, Health and Leisure (CIAFEL), Faculty of Sport, University of Porto, Porto, Portugal; 5 Western Norway University of Applied Sciences, Sogndal, Norway; Mugla Sitki Kocman University: Mugla Sitki Kocman Universitesi, TURKEY

## Abstract

Baecke questionnaire have been widely used to assess physical activity. However, the role of educational level on validity and reliability of Baecke questionnaire is still not stablished, being a factor that can potentially influence the accuracy of self-reported measures. The present study aimed to verify the validity and reliability of Baecke questionnaire for the measurement of physical activity in community dwelling adults according to education level. The sample included 251 adults (42.4±17.0 years, 55% of women). Physical activity was self-reported by Baecke questionnaire and objectively measured by accelerometer. The education level (EL) was classified by years of study into low (<8 years), medium (8–11 years) and high (>11 years). A 7-day test-retest reliability was analyzed by intraclass correlation coefficient. The relationship, agreement and validity of the Baecke questionnaire against accelerometry were analyzed by Spearman’s correlation, Kappa index, and ROC curve, respectively. The reliability of Baecke questionnaire were r = 0.97 (high EL), r = 0.78 (medium EL), and r = 0.68 (low EL). Sensitivity and specificity were 77% and 71% in high EL, 54% and 80% in medium EL and 33% and 89% in the low EL. Baecke questionnaire proved to be reliable and a valid measurement of habitual physical activity in adults with medium and high EL.

## Introduction

Physical activity assessments have been widely used to identify physical activity profiles in different populations and the questionnaire developed by Baecke et al. has been one of the most used questionnaires to assess physical activity level [1], being adopted in both in healthy participants and among those with chronic disease or comorbidities for a wide context of health outcomes [2–5]. This instrument assesses physical activity in different domains (work/occupational activities, sports at leisure time and active commuting) providing a dimensionless score, which corresponds to a specific value where no metric is assigned, such as minutes, metabolic equivalent, or counts. The sum of the three domains provide a total physical activity score which has been used in previous epidemiological studies [6–9]. Total physical activity obtained with the Baecke questionnaire presented a moderate correlation with other methods to measure physical activity [10–12].

The correlations between accelerometer and physical activity questionnaires have been significant regardless of sex and age [13]. The accelerometer is a light and portable device that captures movements in a three-dimensional way and has been widely used to assess physical activity in clinical and epidemiological research. However, this method is limited in practice due to their high cost, data processing, calibration techniques, and output analyses [14]. For these reasons, the use of self-reported methods is a low-cost alternative which could provide large information, which is susceptible to limited accuracy and bias of memory and classification of intensity, mainly in habitual practice and free-living conditions [15].

Among self-reported instruments for the assessment of physical activity levels, a recent systematic review reported that Baecke questionnaire showed better scorings for methodological quality, quality criteria, and high level of evidence than International Physical Activity Questionnaire (IPAQ) [16]. The Baecke questionnaire and the IPAQ were the only questionnaires that considered five domains of physical activity (leisure-time, sports practice, occupational, transportation, and household) [16]. Although IPAQ is recommended for monitoring populational levels of physical activity [17], the time-frame recall of IPAQ corresponds only to the last 7 days, which could affect a habitual real-life estimation, being reported an overestimation of self-reported time spent in physical activities, with a low sensitivity (45%) to detect those who meet physical activity guidelines [18]. In this sense, the Baecke questionnaire is a multiple recording tool for the past 12 months, which is easy to understand and apply, covering qualitative and quantitative indexes, besides addressing a variety of physical activity dimensions with a valid and reliable measurement in Brazilian adults [19]. In addition, Baecke questionnaire was previously validated against gold-standard methods such as doubly labelled water and showed the highest correlation coefficient (r = 0.69, p<0.001) [20].

Previous studies observed that IPAQ–one of the most used self-reported instrument for assessing physical activity–was less valid in participants with low educational level [21, 22]. In addition, Physical activity amounts showed significant difference according to educational levels in accelerometer-measured physical activity of adults [23]. Considering that the Baecke questionnaire involves the report of a wide range of activities, a longer period of time, effort perception, and comparison with other people with the same age, it is possible that education level also affected the validity of the results from this instrument when compared to accelerometry.

The educational level is an indicator of inequality, social problems, and life aspects, such as physical and mental health, family, work, income and environment [24], which may affect the understanding ability of participants and consequently the quality of information provided by the instrument. A positive association was observed between educational level and cognitive function [25], which was inversely associated with precision, validity and lower item nonresponse in health surveys [26]. Furthermore, a better psychical status was associated with more concordant reporting and less underreporting health outcomes [27], whereas education is an indicator of skills needed to acquire social, psychological and economic resources, being considered as the strongest and most consistent predictor of good health than other constructs of socioeconomic status, such as income or occupation, even being interrelated [28]. The knowledge about validity and reliability according to educational level is important to support the development of easier questions and to recognize the limitation of widely used instruments, by considering their bias for specific populations.

Once people with higher educational levels have better knowledge about the practice of physical activity [29], we hypothesized that accuracy of recall may vary according to educational level. However, this information about Baecke questionnaire is not available in literature, so that this investigation is necessary to identify a possible bias when using this widely adopted instrument in community dwelling adults and minimize inferences that educational-related disparities in physical activity levels [30] can be also attributed in part to an assessment bias. Thus, the objective of present study was to analyze the validity and reproducibility of the Baecke questionnaire for the measurement of physical activity in community dwelling adults according to educational level using accelerometry as a guided reference.

## Materials and methods

### Study design and population

This study included a representative sample of adults (≥18 years) from a city located in the southeastern region of Brazil. For the data collection, an accelerometer was delivered for each subject who agreed to participate in the research. After seven days, the researchers returned to the household of the participant to collect the device and schedule the assessments in the next days. The questionnaires were administered in a face-to-face interview.

The sample consisted of 251 adults (138 women) from the city of Santo Anastácio, located in the southeastern region of Brazil (Human Development Index = 0.753). This city has 34 census tracts, of which 23 are located in the urban region and were selected to participate in the present study. In each census tract, the proportionality of inhabitants according to the sample size was considered, where the streets, blocks and households were randomly selected. The research protocol has been previously published [31].

For the sample calculation, a correlation value of r = 0.26 was considered, with an alpha error of 5% and statistical power of 80% [32]. At first, a minimum sample of 125 participants would be required. However, predicting possible sample losses due to the wrong use of the accelerometer, the sample was added 50% more, which generated a minimum total number of 188 participants to be evaluated. All the participants invited and who agreed to participate in this research signed a written consent form. The study was approved by the Ethical in Research Committee from Sao Paulo State University (UNESP) at protocol CAAE 72191717.9.0000.5402.

### Education level

The education level of participants was stratified according to self-reported years of schooling. A question about education level was included in the survey as follows: “What is your education level?” Responses were: i. illiterate or incomplete primary school (less than 4 years of schooling); ii. complete primary school or incomplete elementary school (from 4 to 7 years of schooling); iii. complete elementary school or incomplete high school (from 8 to 11 years of schooling); iv. complete high school or college (12 years of schooling or more). Participants with less than 8 years of schooling were classified as low education level; participants between 8–11 years of schooling were classified as medium education level; and those participants with more than 11 years of schooling were classified as high education level.

### Questionnaire-assessed physical activity

The physical activity questionnaire used in the present study was proposed by Baecke et al. and was previously published in the literature [1], being previously validated in Brazilian adult population [19]. This questionnaire was used to assess physical activity in three different domains: physical activity at work, sports practice, and leisure time/commuting physical activities, and provides a dimensionless score for habitual practice of physical activity [1]. The internal consistency of Baecke questionnaire was previously analyzed by Florindo et al. [33], which reported a Cronbach a ranging from 0.52 to 0.62 across the three assessed domains. This instrument is composed by 16 questions scored in a Likert scale from 1 to 5 for each question, where a specific formula provide a score between 1 and 5 for each assessed domain, with a total physical activity score ranging from 3 to 15 (by the sum of three domains score) [1]. This dimensionless score is a specific value which does not correspond to duration, intensity, or energy expenditure indicator, and does not have cutoff points to define who is physically active.

In a subsample of 50 participants the temporal stability was analyzed applying the questionnaire after one week using the same procedures employed in the first visit.

### Accelerometer-assessed physical activity

An objective measure of physical activity was collected using an Actigraph GT3X tri-axial accelerometer (ActiGraph, LLC, Pensacola, FL, USA) capable of recording movements in the three orthogonal planes: vertical, anteroposterior horizontal and mid-lateral. This instrument is a small, light and non-invasive device that is used by participants during waking hours for five consecutive days on the right hip. The whole day acceleration data was sampled at 30Hz and analyzed in a specific time interval of 60 seconds (1-min epoch). This pattern of time interval was used for being the closest to the pattern of long duration physical activity, due to the ease of conversion from activity counts into minutes of activity [34] and the possibility to use validated cutoff points in counts per minute for classification of physical activity intensity. A complete data was defined as having at least 10 hours of daily use monitored for four days [34, 35]. We defined as non-wear time those periods of at least 60 consecutive minutes from zero counts, with an activity interruption permission from 0 to 100 counts/minutes and a maximum duration of two consecutive minutes [36].

The measures of physical activity derived from the accelerometer were i) sum of moderate and vigorous physical activity per week. The minutes per week estimate is based on the Troiano cutoff point [35]: moderate intensity: 2020–5998 counts; and for vigorous intensity: 5999 counts or more. The accelerometer variables were obtained from the vertical axis. The data collected with the accelerometer were analyzed using the Actilife 6 software (ActiGraph, LLC, Pensacola, FL, USA).

### Statistical analysis

The data normality was verified by the Shapiro-Wilks test. Since normality was not detected, the sample characterization variables were shown in median and interquartile range. The median comparison according to the three groups (high, medium and low education level) was verified by the Kruskal-Wallis test, with differences identified by the Mann–Whitney U test. The temporal stability of data was analyzed by intraclass correlation coefficient (ICC), while the relationship between physical activity measured by the questionnaire and accelerometer was verified by Spearman’s correlation (continuous analysis).

The participants were further classified into quartiles of physical activity, where participants located in the fourth quartile of the Baecke questionnaire and of the measure of accelerometer were considered as more active, while the participants located in the lower quartiles were considered as less active. This classification of Baecke score was used due to the lack of cutoff point of this instrument to define physical activity level, as adopted in several studies in literature [8, 37–39]. The agreement between the methods was verified by the Kappa coefficient. This categorization was performed so that the sensitivity and specificity analysis determined by the Roc Curve could be tested, where the accelerometer is the gold standard method for physical activity and the Baecke questionnaire was the indirect measure to be tested. The statistical significance adopted was 5% and the confidence interval was 95%, with analysis performed by IBM SPSS Statistical Package, version 25.

## Results

The final sample of 251 adults were composed by 55.0% of women (n = 138) and presented an average age of 42.4±17.0 years. Regarding educational level, the proportion of participants in each group was 13.5% of low education level (n = 34), 58.2% of medium educational level (n = 146), and 28.3% of high educational level (n = 71). The characteristics of the sample are shown in **Table 1**. Participants from the low educational level were older and had shorter stature compared to participants with medium and high educational level. The intraclass correlation coefficient of the Baecke questionnaire was ICC = 0.72 (p<0.001) when considered the whole sample, ICC = 0.67 (p<0.001) for participants with low educational levels, ICC = 0.75 (p = 0.010) for participants with medium educational level and ICC = 0.97 (p = 0.004) for those with high educational level.

**Table 1:** Sample characteristics (n = 251).

	Whole sample		High EL		Medium EL		Low EL		
Variables	Median	IQR	Median	IQR	Median	IQR	Median	IQR	p-value
Age, y	42.0	27.0–55.0	37.5	27.0–49.0	42.0	22.0–53.0	64.0^a^	53.0–71.0	<0.001
Weight, kg	75.6	64.5–86.4	79.7	67.4–88.5	75.3	64.0–85.7	73.6	63.5–84.1	0.125
Stature, m	1.65	1.59–1.73	1.68	1.62–1.76	1.65	1.58–1.73	1.58^a,b^	1.52–1.65	<0.001
Questionnaire PA, Baecke score	7.6	6.7–8.8	8.1	7.0–9.0	7.6	6.7–8.7	7.0^a^	5.4–7.9	0.008
Accelerometry PA, min./week	178.0	99.8–282.6	183.9	179.2–249.0	186.0	100.7–303.0	162.1	67.5–293.4	0.625

EL: Education level; IQR: Interquartile range (P25-P75); PA: Physical activity; a: difference with High EL; b = difference with Medium EL.

**Fig 1:**
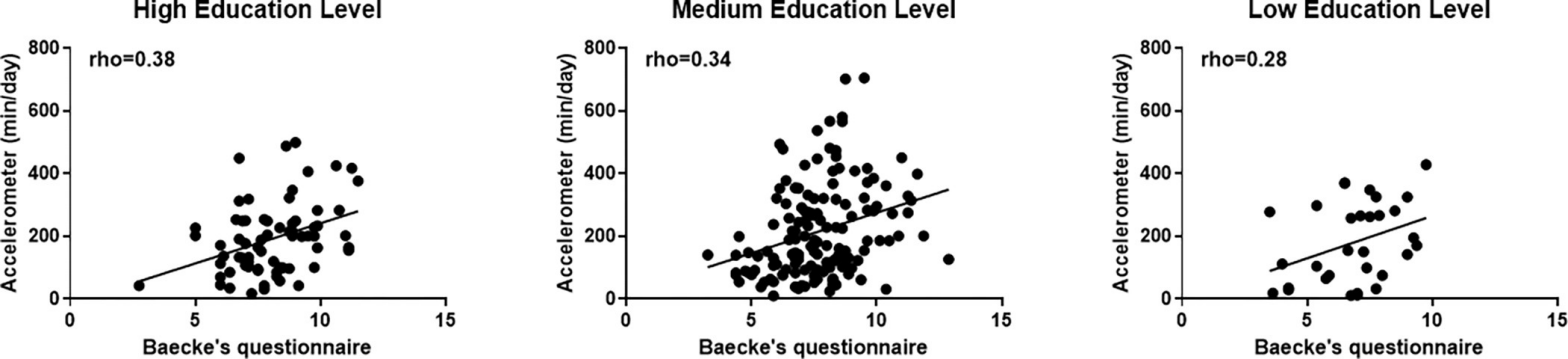
Correlation between Baecke questionnaire and physical activity assessed by accelerometer.

**Fig 1** shows the correlations between physical activity measured by the Baecke questionnaire and accelerometer. According to education level, the correlations were significant in high and medium educational levels, with a decrease in coefficient as the education level decreases, loosing significance among participants with low educational level. Spearman correlation coefficient were rho = 0.38 (p<0.01) in participants with high education level, rho = 0.34 (p<0.01) in participants with medium physical educational level, and rho = 0.28 (p = 0.117) in participants with low educational level.

The bivariate correlation matrix between this study variables is presented in the **Table 2**. The age of participants was higher in females (rho = 0.13; p = 0.030) while was inversely correlated with education level (rho = -0.29; p<0.001), leisure-time physical activity (rho = -0.17; p = 0.05), sports practice (rho = -0.19; p = 0.002), total Baecke score (rho = -0.14; p = 0.023) and physical activity by accelerometry (rho = -0.23; p<0.001). Considering men as reference, females presented lower leisure-time physical activity (rho = -0.15; p = 0.012), sports practice (rho = -0.13; p = 0.032) and physical activity by accelerometry (rho = -0.15; p = 0.018). Education level was positively correlated with sports practice (rho = 0.31; p<0.001) and total Baecke score (rho = 0.18; p = 0.004). Among the physical activity domain scores, only sports practice and occupational score were not mutually correlated (rho = 0.03; p = 0.590). Total Baecke score and accelerometry were significantly correlated (rho = 0.33; p<0.001).

**Table 2:** Correlation matrix between variables (n = 251).

	Spearman correlation coefficient (rho)						
Age	Age						
Sex (men as reference)	0.13*	Sex (men as reference)					
Education level	-0.29**	-0.04	Education level				
Leisure-time score	-0.17**	-0.15*	0.08	Leisure-time score			
Sports practice score	-0.19**	-0.13*	0.31**	0.34**	Sports practice score		
Occupational score	0.06	0.05	-0.02	0.13*	0.03	Occupational score	
Total Baecke score	-0.14*	-0.10	0.18**	0.66**	0.63**	0.65**	Total Baecke score
Accelerometry	-0.23**	-0.15*	-0.01	0.25**	0.26**	0.17**	0.33**

*p-value <0.05

**p-value <0.01.

**Table 3** shows the sensitivity and specificity of the Baecke physical activity questionnaire and accelerometer. The accuracy and sensitivity of questionnaire were higher with increases in educational level. Specificity had good values at all educational levels (> 0.70), being higher in participants with low education level.

**Table 3:** Analysis of accuracy, sensitivity and specificity of the Baecke questionnaire to assess physical activity against the accelerometry method.

	Accuracy (95% CI)	Sensivity (95% CI)	Specificity (95%CI)
Whole sample	69.2 (63.1–74.9)	59.4 (46.4–71.2)	79.0 (72.5–84.6)
High EL	73.8 (62.0–83.5)	76.9 (46.2–95.0)	70.7 (57.3–81.9)
Medium EL	67.1 (58.8–74.7)	53.7 (37.4–69.3)	80.6 (71.6–87.7)
Low EL	60.9 (43.0–76.9)	33.3 (7.5–70.0)	88.5 (69.8–97.6)

EL = Education level; 95%CI = Confidence Interval.

## Discussion

The present study observed a good temporal stability in a 7-day test-retest of Baecke questionnaire, being increased as the education level was higher. The correlation between Baecke questionnaire and accelerometry were significant in whole sample, but was not confirmed among participants with low educational level. The education level was positively correlated with Baecke scores, but not with accelerometry. Baecke questionnaire showed good validity in community dwelling adults when compared to accelerometry, with better accuracy and sensitivity among participants with medium-to-high education level.

The Baecke questionnaire showed good temporal stability in the present study (ICC = 0.72), but this value was lower than previous studies in literature: Ono et al. [11] observed an ICC = 0.87 in a sample of 61 adult women; Stefanouli et al. [40] reported an ICC = 0.84 among 30 Greek healthy adults; and Sadeghisani et al. [41] reported an ICC = 0.88 when assessed 32 healthy Persian adults. The present study was composed by a larger adult sample randomly recruited in the community, which may justify higher inconsistencies in Baecke questionnaire when compared to specific populations or limited sample sizes. Besides that, the present study observed that ICC values increased as the education level was higher, reaching ICC = 0.97 among participants with high educational level.

The present study observed a significant correlation between Baecke questionnaire and accelerometry, which was not confirmed only among participants with low education. Philippaerts et al. [12] reported a significant correlation coefficient of r = 0.47 between Baecke questionnaire and a 4-day movement registration from a tri-axial accelerometer. Mahoney & Freedson [42] reported a correlation of r = 0.53 between Baecke questionnaire and 4-day registration of Caltrac® accelerometer, which was not confirmed by Miller et al. [43], in a study involving 35 physical therapists. More recent studies analyzing the correlation between Baecke questionnaire and accelerometry were not found in literature, neither previous study involving Brazilian adult population.

Previous studies which analyzed correlation of IPAQ against accelerometry also observed limitations among participants with lower educational levels [21, 22]. Dyrstad et al. [21] in a study with adults aged 17–84 years, observed that participants reported more minutes of vigorous physical activity by IPAQ Short-form questionnaire when compared to accelerometer, with greatest difference observed among lower education level group. Winckers et al. [22], in a study with 196 healthy adults, reported that physical activity assessed by IPAQ was less valid against accelerometer in those participants with low education level, where Spearman rho ranged from -0.07 to 0.05 in this group vs. 0.16 to 0.27 in overall sample.

The present study observed a medium sensitivity of Baecke questionnaire for identifying most active participants when considering the whole sample (59.4%). However, this sensitivity was higher among those with medium and high educational level, being very weak in group with low education level (33.3%). Otherwise, all education level groups showed a good specificity to detect those participants who were less active (≥70%). The reasons for the lower validity in participants with lower educational level included difficulties in capturing manual physical activities by the accelerometer and difficulties in the interpretation of some sections of the questionnaire [44]. It is also important to highlight that the Baecke questionnaire has a different construct than IPAQ, which considers duration, intensity, weekly frequency, and for how long these activities are habitually performed in a 12-month recall. These questions may be more susceptible to bias of memory and classification of intensity by the participants, as well as the perception to compare with the activity performed by their peers (as asked in some questions of instrument). These factors can be dependent of education level of participant to be more accurate, once a clearly perception and detailing are need.

Regarding the bivariate correlation between study variables, it was observed an inverse correlation of physical activity with age (with only exception of occupational score) and with female gender (leisure time, sports practice, and accelerometry). These findings were convergent with previous studies, once Speakman & Westerterp [45] observed that physical activity decreased as the age increased, and Ayabe et al. [46] reported that the decline in physical activity with age occurred in those with moderate-to-vigorous intensity. Li et al. [47] reported that greater decline in physical activity with age was observed among women, which also presented lower physical activity level than men, mainly regarding those activities with moderate-to-vigorous intensity. Otherwise, the present study observed that education level was positively correlated with Baecke questionnaire, but not with accelerometry. It is possible that time-frame recall of 12-month form Baecke questionnaire reaches a more detailed subjective parameters of physical activity behaviors than short-term accelerometry counts, which could justify this inconsistency between methods (habitual 12-month practice vs. 5-day recording).

When considered the distribution of sample according to education level, this study observed that participants with low education level were older than those with medium and high education level. A recent report of Brazilian Institute of Geography and Statistics revealed that as higher the age group, higher is the proportion of illiterates, reaching 18.6% of illiterate prevalence among people with 60 years and more [48]. In addition, the age is a great contributor to reduced physical activity level [45, 46] and the lower practice of physical activity may compromise the better identification of this behavior in different domains of daily life.

As limitations of the present study, we considered that all minutes in MVPA counts were equal, even if some are from high intensity and some others only moderate, which could decrease the strength of association. Besides that, the lack of application of IPAQ in the sample precluded comparison between instruments according to educational level against accelerometry. The heterogeneity of age and proportion of participants according to education level is another important limitation point of this study findings, which highlights the need to consider the influence of the age in the comprehension of Baecke questionnaire, with a cautious adoption of a face-to-face application of this questionnaire in older participants, due to the higher age of participants with low education level. Otherwise, strengthen points can be highlighted, such as the representative sample of a Brazilian city randomly selected by census tracts, streets, blocks and households and for being the first study to investigate validity and reliability of Baecke questionnaire against accelerometry according to educational level in community dwelling adults.

In summary, the Baecke questionnaire correspond to an alternative to assess the measure of physical activity on a large scale in community dwelling adults, showing a good reliability and validity for measuring physical activity in participants with medium and high educational level. However, the Baecke questionnaire seems not to be reliable in adults with low education level and those who were elderly, so that cautions needs to be adopted for application in this population. Therefore, the application of the Baecke questionnaire among participants with low educational level and among those who were elderly should be applied in a face-to-face form, so that explanation of questions can be provided to the participants and possible doubts can be solved.
